# Decrease in Salivary Serotonin in Response to Probiotic Supplementation With *Saccharomyces boulardii* in Healthy Volunteers Under Psychological Stress: Secondary Analysis of a Randomized, Double-Blind, Placebo-Controlled Trial

**DOI:** 10.3389/fendo.2021.800023

**Published:** 2022-01-06

**Authors:** Michał Seweryn Karbownik, Joanna Kręczyńska, Anna Wiktorowska-Owczarek, Paulina Kwarta, Magdalena Cybula, Nebojša Stilinović, Tadeusz Pietras, Edward Kowalczyk

**Affiliations:** ^1^ Department of Pharmacology and Toxicology, Medical University of Lodz, Łódź, Poland; ^2^ Department of Infectious Diseases and Hepatology, Medical University of Lodz, Łódź, Poland; ^3^ Psychiatric Ward for Adolescents, Babinski Specialist Psychiatric Healthcare Center, Łódź, Poland; ^4^ Oklahoma Medical Research Foundation, Aging and Metabolism Program, Oklahoma City, OK, United States; ^5^ Department of Pharmacology, Toxicology and Clinical Pharmacology, University of Novi Sad, Novi Sad, Serbia; ^6^ Department of Clinical Pharmacology, Medical University of Lodz, Łódź, Poland

**Keywords:** probiotic, yeast, *Saccharomyces boulardii*, dietary supplementation, salivary serotonin, symapathoadrenal markers, anxiety, depression

## Abstract

**Background:**

Bacterial probiotics are thought to exert a serotonergic effect relevant to their potential antidepressant and pro-cognitive action, but yeast probiotics have not been tested. The aim of the present study was to determine whether 30-day supplementation with *Saccharomyces boulardii* affects the level of salivary serotonin under psychological stress and identify the factors associated with it.

**Methods:**

Healthy medical students were randomized to ingest *Saccharomyces boulardii* CNCM I-1079 or placebo before a stressful event. Salivary serotonin concentration was assessed before and at the end of supplementation. Moreover, obtained results were compared to psychological, biochemical, physiological and sociodemographic study participants data.

**Results:**

Data of thirty-two participants (22.8 ± 1.7 years of age, 16 males) was available for the main analysis. Supplementation with *Saccharomyces boulardii* decreased salivary serotonin concentration under psychological stress by 3.13 (95% CI 0.20 to 6.07) ng/mL, *p* = 0.037, as compared to placebo. Salivary serotonin was positively correlated with salivary metanephrine (β = 0.27, 95% CI 0.02 to 0.52, *p* = 0.031) and pulse rate (β = 0.28, 95% CI 0.05 to 0.50, *p* = 0.018), but insignificantly with anxiety, depression, eating attitudes and information retrieval.

**Conclusions:**

*Saccharomyces boulardii* CNCM I-1079 may be distinct from bacterial probiotics in its salivary serotonergic effect, which appears positively linked to symapathoadrenal markers. The study requires cautious interpretation, and further investigation.

## Introduction

Serotonin (5-hydroxytryptamine, 5-HT) is a ubiquitous monoamine hormone and neurotransmitter that plays a role in numerous biological processes. It is synthesized from an essential amino acid, tryptophan (Trp), and degraded to 5-hydroxyindoleacetic acid (5-HIAA) for removal in the urine (1, 2). In the periphery, 5-HT is produced in the enterochromaffin cells (EC) of the digestive tract and transported to blood platelets. Its role includes stimulation of gastrointestinal peristaltic reflexes, amplification of platelet aggregation, and control of vascular tone and cardiac function (1–5). Although little 5-HT is synthesized and found in the central nervous system (CNS), it nevertheless modulates virtually all behavioral and neuropsychological processes including, but not limited to, mood, cognition, perception, reward, anger, aggression, appetite, sexuality, sleep, and attention (1, 2, 6–8). It also appears to diminish the psychological stress response (9–11).

The gut microbiota is capable of producing and releasing 5-HT and its precursor Trp (12–14) or modulating 5-HT synthesis from human colonic EC (15). Several studies have shown the gut microbes and bacterial probiotic supplementation to exert central and peripheral serotonergic effect. In preclinical settings, colonization of germ-free mice with human gut microbiota restored physiological expression of a colonic 5-HT synthesizing enzyme (16), whereas feeding normal rats with *Bifidobacterium infantis* increased plasma Trp concentration and decreased frontal cortex 5-HIAA level (17). In the clinical settings of randomized controlled trials, dietary supplementation with fermented milk containing *Lacticaseibacillus casei* strain Shirota resulted in a time-specific increase in fecal 5-HT and a decrease in plasma Trp in healthy volunteers under psychological stress (18). Consumption of *Lactiplantibacillus plantarum* DR7 by stressed human adults, reduced anxiety symptoms and enhanced the 5-HT pathway, as observed by its modified plasma metabolic enzyme expression profile (19). In addition, consumption of a mixture of *Lactobacillus helveticus* and *Bifidobacterium longum* by patients with depression resulted in mood improvement linked to an increase in relative serum Trp level, which implied enhanced 5-HT synthesis (20). It has been proposed that improved availability of 5-HT is a mediator of probiotic-induced antidepressant and pro-cognitive effects (21–23).

In humans, among many biological fluids, 5-HT is also present in saliva (24–27). It appears to derive from two 5-HT pools, one associated with the CNS and another with the periphery (28, 29), both providing some contribution. Changes in diurnal rhythm amplitude in salivary 5-HT of depressed patients treated with a selective serotonin re-uptake inhibitor were positively correlated with improvement in depressive symptoms (27). Social sharing of happiness and empathic abilities decreased as a function of salivary 5-HT level (26). These suggest that salivary 5-HT is CNS-derived. On the other hand, 5-HT level in saliva was found to be only weakly and inconsistently correlated with its level in cerebrospinal fluid (CSF) in children presenting with neurological symptoms (24). In adult phenylketonuria patients, no association was found between the concentration of 5-HT in saliva and its turnover in the CNS (25). Salivary 5-HT concentration was not related to post-exercise euphoria (30). Also, although it has been proposed that central serotonergic nervous system activity decreases salivary cortisol reactivity under stressful conditions, no such correlation was observed between salivary 5-HT and cortisol in dogs (31). This body of evidence, in turn, supports the peripheral origin of 5-HT in saliva.

Interestingly, salivary 5-HT was proposed to reflect the gut microbiota status. In a recent study, dogs were supplemented with grapevine-extracted proanthocyanidins or placebo; both intervention groups demonstrated an increase in salivary 5-HT, which was attributed to a relative abundance of fecal *Escherichia coli* (31). Thus, saliva appears to be a suitable source of 5-HT to study serotonergic modulation induced by the gut microbiota.

Although most probiotics are bacteria, the yeast *Saccharomyces cerevisiae* var. *boulardii* (*Saccharomyces boulardii*, *Sb*) has also been found to have probiotic properties (32). *Sb* has been extensively tested and widely used in gastroenterology (32, 33); however, it is a subject of very little research in neuroscience (34, 35), particularly in relation to its serotonergic effect. Intriguingly, fungal probiotics significantly differ from bacterial ones in their biological characteristics, mechanism of action and beneficial properties (36, 37), and the effects of probiotic bacteria cannot be easily extrapolated to yeasts (35). Therefore, there is a need to determine the serotonergic effect of yeast probiotics and its potential therapeutic implications.

The primary aim of the present study was to assess whether healthy volunteers under psychological stress demonstrate alterations in salivary 5-HT concentration when receiving a yeast probiotic, *Sb* strain CNCM I-1079, for 30 days as a dietary supplement, in comparison to placebo. The secondary aim was to test whether salivary 5-HT concentration is associated with selected psychological (depression, anxiety, eating attitudes, information retrieval), physiological (pulse rate, body-mass index), biochemical (salivary cortisol and metanephrine) and sociodemographic parameters. The study serves as a secondary analysis of a randomized, double-blind, placebo-controlled trial aimed at evaluating the pro-cognitive and anti-anxiety effects of *Sb* dietary supplementation (original study) (35).

## Materials and Methods

### Study Design

The present study is a secondary analysis of a unicenter, parallel-group, three-arm, 1:1:1 allocation ratio, randomized, double-blind, placebo-controlled, superiority trial (original study) (35). It comprised two intervention arms, one with *Sb* CNCM I-1079 strain and the other with *Lacticaseibacillus rhamnosus GG* ATCC 53103 (previously *Lactobacillus rhamnosus*, *Lr*) strain, as well as a placebo comparator containing a mixture of maltodextrins in the form of oral capsules. The tested products were administered for 30 days to three groups of randomly allocated healthy medical students. Following this, they underwent a stressful event modeled by an academic examination [the presence of psychological stress accompanying this event was manifested by increased salivary cortisol level and resting pulse rate, and enhanced state of anxiety (35)]. Sociodemographic, basal psychometric and some other data was provided at the entrance to the study, whereas state anxiety and pulse rate were assessed twice: before supplementation, “at rest”, and at the end of supplementation, “under stress”. Similarly, salivary samples were collected twice: once “at rest” and once “under stress”. The product containing *Lr* was found to be degraded in the quality control test performed after the trial completion (35). As a result, no analysis of the *Lr* arm was performed in the present study. The methods that are common for the present and original study (35) have been described here in brief, and are given in more detail in the original study report (35).

### Ethical Considerations

The Deans of the Faculty of Medicine and Faculty of Military Medicine as well as the Bioethics Committee of the Medical University of Lodz approved the original study (RNN/86/16/KE, received on 19 April 2016). The Bioethics Committee approval allowed for 5-HT level assessment; however, it was not included to the protocol of the original study (U.S. National Institutes of Health https://clinicaltrials.gov, NCT03427515, retrospectively registered on 09 February 2018), and thus the present study was considered its secondary analysis (38, 39). Written informed consent was obtained from all participants upon entrance to the study.

### Participants

Healthy voluntary medical students were recruited. The inclusion criteria were as follows: being a third-year medical student of the Faculty of Medicine or Faculty of Military Medicine (Medical University of Lodz, Poland) and age 18-30 years. Exclusion criteria included:

- formal inability to sit the first attempt of the final examination in Pharmacology, which served as a model of psychological stress
- chronic diseases: neurological, psychiatric, cardiological, gastroenterological, immunological, endocrine, infectious or state of immunosuppression
- history of hospitalization (up to three months before entrance to the study), presence of central venous catheter or parenteral nutrition
- current pregnancy, intention to become pregnant within three months from the entrance to the study or current lactation
- allergic reaction (up to three months before entrance to the study)
- hypersensitivity to any ingredients of the supplements (yeast, maltodextrins, potato starch, magnesium stearate, hypromellose, gelatin, glycerol, or titanium dioxide)
- body-mass index over 30
- chronic medication use (up to three months before entrance to the study; “chronic” was defined from a frequency perspective as “at least 90 days a year on average”; pharmacological contraceptives were not considered “medication” and were allowed in the study)
- systemic antibacterial or antifungal medication use (up to three months before entrance to the study)
- overuse of alcohol [defined as 20 g and 40 g of pure ethanol *per* day for females and males, respectively (40)] or any psychoactive substances (up to three months before entrance to the study)
- smoking more than five cigarettes (or equivalent) a day (up to three months before entrance to the study)
- pro- or prebiotic preparations intake (up to three months before entrance to the study)
- vegan or other atypical diet

The model of repeated measures analysis of variance (ANOVA) for within-between interaction was used to determine the required minimum sample size. A statistical power of β = 0.8 was set. As the estimation referred to no preliminary results, a medium effect size of Cohen’s *f* = 0.25 and default correlation among repeated measures of *r* = 0.5 were assumed. The sample size was determined to be at least 17 in each study group. G*Power software version 3.1.9.2 was used for sample size estimation (41).

### Intervention and Control Product

The dietary supplement preparation *LacidoEnter* was purchased (Institut Rosell; Montreal, Canada; batch numbers HG09241 and HI17731; expiry date 01/2017 and 03/2017, respectively). *LacidoEnter* is commercially-available in Poland. The product was declared to contain lyophilized *Sb* CNCM I-1079 in an amount of 5×10^9^ colony forming units (CFU) *per* dose. The content of the original capsules were transferred to new gelatin capsules (ACG Associated Capsules; Maharashtra, India) and filled with a mixture of maltodextrins (Pepees; Łomża, Poland) *quantum satis*. Placebo products were obtained by filling empty capsules with the mixture of maltodextrins only. The choice of maltodextrins was dictated by their organoleptic properties mimicking lyophilized probiotic powder. The formulations were prepared using Capsunorm, a manual capsule filling device (Eprus; Bielsko-Biała, Poland).

The quality control test performed after the trial completion revealed adequate number of *Sb* CFU *per* dose in the intervention product [2.1×10^9^, 95% confidence intervals (CI) 0.8×10^9^ to 5.4×10^9^, which is above the typically recommended daily dose (33)]. Placebo product exhibited no *Sb* CFU *per* dose.

### Procedure

**Figure 1:**
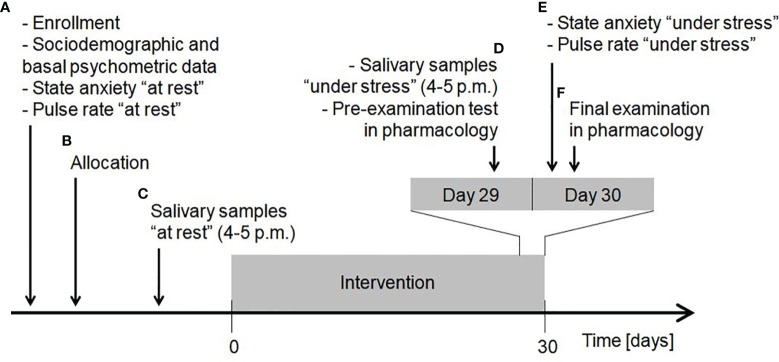
Timeline of the study procedures. Explanation in the text. Reproduced from (35).

The original study was carried out between 20 April and 20 June 2016. Third year medical students of the Faculty of Medicine and Faculty of Military Medicine, Medical University of Lodz, Poland, were invited to participate. At the study inaugural session (**Figure 1**, point A) written informed consent was obtained from all the participants, who then drew random paper sheets with printed numbers (participant codes) to blind all the samples and questionnaires throughout the study. During this session, sociodemographic, basal psychometric and some other data was collected, state anxiety “at rest” was examined and pulse rate “at rest” was self-recorded for 30 seconds by palpation at the radial artery after at least 10-min obligatory rest in a sitting position. Psychometric questionnaires included Polish versions of the Eating Attitudes Test-26 to measure the symptoms and concerns characteristic of eating disorders (42, 43), the Beck Depression Inventory to measure severity of depressive symptoms (44, 45), Perceived Stress Scale-10 to measure the extent of perceived stress (46, 47) and State-Trait Anxiety Inventory (STAI) to assess both state and trait anxiety (48, 49). Afterwards, a process of simple randomization using participant codes was performed to obtain group allocation with the same probability of being assigned to each study arm (**Figure 1**, point B).

**Figure 2:**
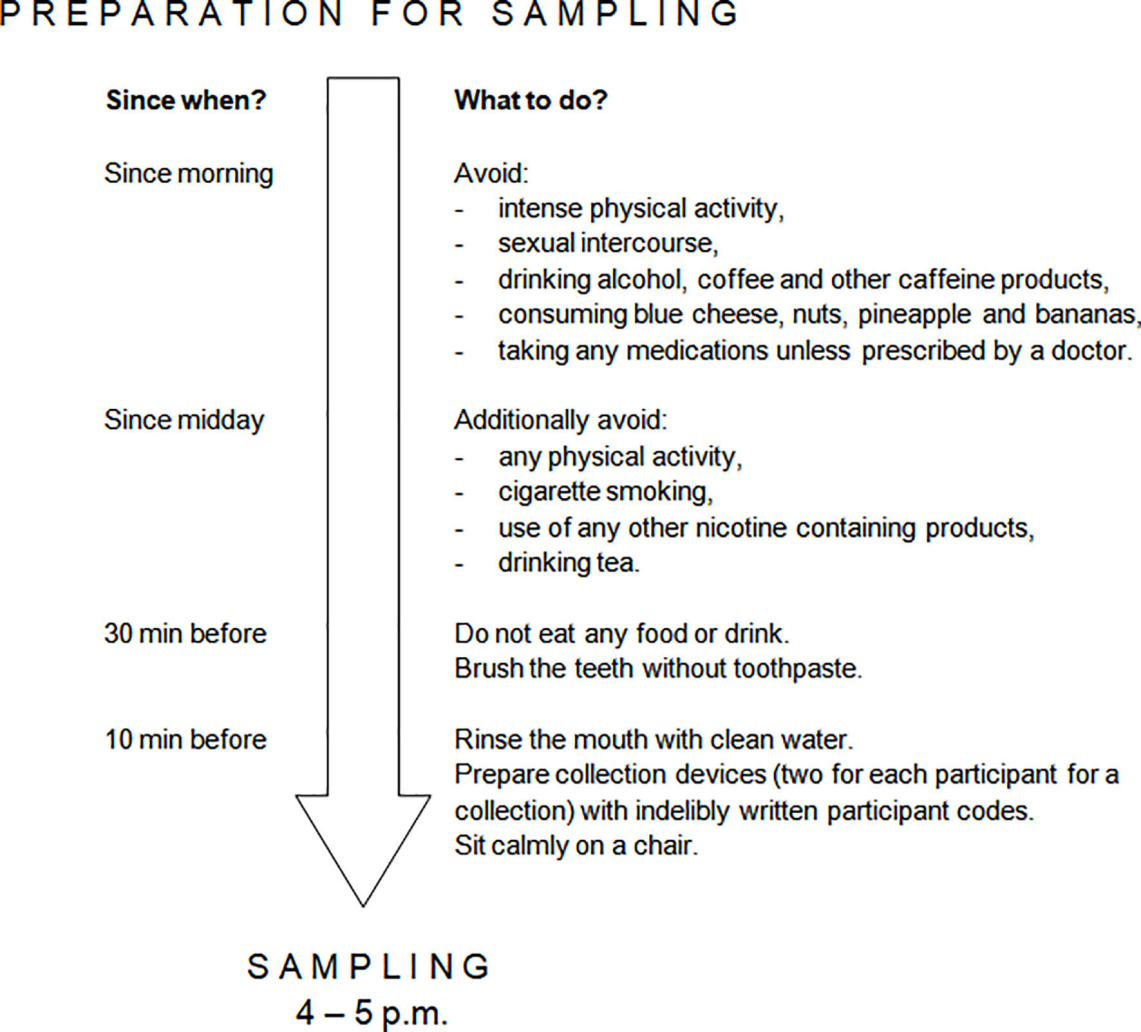
Requirements for salivary sample self-collection. Preparation was performed in the day of salivary collection. Each participant was provided with detailed instruction on how to prepare to and perform salivary sample self-collection. The need to avoid nuts, pineapple and bananas was due to the presence of serotonin or tryptophan in these products (50, 51).

Salivary samples “at rest” were self-collected at home (**Figure 1**, point C). The participants were instructed to perform salivary sampling at a chosen non-stressful time before the intervention started. Saliva was collected by chewing Salivette Cortisol polyethylene swab (Sarstedt; Numbrecht, Germany) in a seated position between 4 p.m. and 5 p.m., when maximum diurnal level of salivary 5-HT is present in healthy people and thus little variation occurs (27). Participants required rigorous preparation on the day of sample collection (**Figure 2**). The obtained saliva samples were stored in the domestic refrigerator and transported to the Department of Pharmacology and Toxicology, Medical University of Lodz on ice within 24 hours from specimen collection, where they were immediately frozen at -20°C until analysis. When depositing salivary samples in the Department, the participant received packaging of dietary supplement product marked with participant code and a leaflet attached. The participants were instructed to take one capsule a day (enough to constantly function in the gut due to *Sb* residence time of 2-5 days (52)), swallowed as a whole in the morning during or after the breakfast and to store the supplement at room temperature in a dry and dark place throughout the study.

At the end of the intervention, a day before the final examination in Pharmacology, the participants self-collected salivary samples “under stress” (Day 29; **Figure 1**, point D). This was performed under the same restrictions (**Figure 2**) and with the same procedure as previously described in “at rest” time point, and the samples were stored and transported according to the same conditions. On that day, participants also performed an online pre-examination test in Pharmacology (SurveyMonkey; San Mateo, CA, USA), which consisted of 30 yes/no questions (one point for each correctly answered). The test served as a comparator for the results of formal examination (to adjust statistical analysis of final exam score to a pre-examination test score). Just before the final examination in Pharmacology (Day 30; **Figure 1**, point E), in the examination building, participants were asked to complete state anxiety STAI test and self-record their pulse rate “under stress” according to the same protocol as “at rest”. They took the final exam (**Figure 1**, point F), which consisted of 60 computer-based multiple choice questions. The exam was performed in three rounds and examination set was included as a grouping factor in statistical analysis.

### Salivary Concentration of 5-HT and Other Measurands

For the purpose of this study only salivary samples of *Sb* and placebo groups were analyzed. Immediately after being thawed, the samples were centrifuged at 1000 g in 4°C for 5 min to recover saliva from a swab. The supernatants were transferred to new vials kept on ice. No apparent blood contamination was visible in any of the tested samples. 5-HT concentration was determined in salivary supernatants by an enzyme-linked immunosorbent assay (ELISA) with the use of Serotonin Research ELISA DEE5900 kit (Demeditec Diagnostics; Kiel, Germany), which is dedicated for any biological sample. Cross-reactivity of the assay was reported by the producer to be 0.19% for tryptamine, 0.03% for melatonin and less than 0.002% for 5-HIAA.

Before analysis, each salivary supernatant was diluted four times with an attached diluent. Two technical replicates were run for each sample. The 5-HT within the salivary samples was subjected to acylation and then detected by competitive ELISA in a microtiter plate format. A BioTek EL ×800 microplate reader (BioTek; Winooski, VN, USA) was used to measure absorbance at 450 nm with a reference of 630 nm. A four-parameter logistic model was used to plot a calibration curve. The additional concentration point was added to the calibration curve (five times higher than the most concentrated). This, together with salivary supernatant dilution, was applied to increase the upper limit of quantification (ULOQ) for 5-HT to 50 ng/mL to capture the highest expected levels present in saliva (26, 27). Including an additional point in the calibration curve was justifiable as the intra-assay coefficients of variation (CVs) for the highest concentrations were acceptably low (53). The experimentally set intra- and inter-assay CVs for all the samples were 12.3% and 18.3%, respectively and were minimally higher than that reported in some other salivary 5-HT research (26, 31). Out of 64 salivary samples, five (8%) 5-HT concentrations were above ULOQ; these were replaced by a value of 50 ng/mL.

Between the experiments to determine 5-HT concentrations, an unnoticed electrical network failure occurred in the laboratory building. This caused the freezers to turn off and some remaining salivary samples to thaw out. As the thawed samples stayed at room temperature for several hours, they were considered degraded. These samples were not subjected to 5-HT concentration assessment. The missing 5-HT data was assumed *missing completely at random* as the 5-HT ELISA analyses had been performed in the order of participant codes, which were drawn randomly (54). The problem of sample degradation also prevented reanalysis of the samples with 5-HT levels above ULOQ and the samples with inconsistent results presenting high CVs.

### Data Analysis

The main analyses were performed *per protocol* (PP), *i.e.* including all participants for whom salivary 5-HT concentration was actually measured. If the main analysis returned statistically significant result, two *intention to treat* (ITT) sensitivity analyses were additionally performed: modified ITT (mITT), which included all the subjects who completed their assigned supplements and true ITT (tITT), which included all the randomized subjects with their original group allocation (55). For the purpose of ITT analyses, the missing salivary 5-HT concentration data was filled in using multiple imputation by chained equation procedure.

Before the analyses started, salivary 5-HT concentration was log-transformed to bring the distribution closer to normal. A two-way repeated-measures analysis of variance (ANOVA) model was applied to achieve the primary aim of the study. The between-subject factor was the study group, whereas the within-subject factors were the “at rest” and “under stress” time points. The within-between two-way interaction was evaluated to determine whether the difference in salivary 5-HT concentration between the “at rest” and “under stress” states varies between the study groups. Adjusting for highly imbalanced baseline parameters was considered (56). In addition, one-way repeated-measures ANOVA was performed to evaluate whether psychological stress affects salivary 5-HT concentration separately in both groups. The results were expressed as the mean difference in salivary 5-HT concentration with 95% CI.

General linear modeling procedures were applied for the secondary aim of the study. It was tested whether the “at rest” and “under stress” salivary 5-HT concentration values correlated with the available sociodemographic, psychological, biochemical and physiological characteristics of the study participants. For state anxiety, salivary cortisol and metanephrine, and pulse rate, the study data for both time points were combined to increase statistical power, assuming no difference in extent of the correlation between “at rest” and “under stress” conditions; these variables had separate “at rest” and “under stress” data pairs with salivary 5-HT. Additional parameters were included to some of these analyses to control the potential effect of confounders. Parametric analyses were applied, although some of the variables were ordinal. This was to allow more flexible modeling and was justifiable as such analyses are usually closely compatible with non-parametric ones (57). While testing multiple hypotheses, Benjamini and Hochberg procedure was used to control the false discovery rate at the level of 0.3. Such value was justified by exploratory nature of the correlation analyses.

A *p*-value lower than 0.05 in the main analyses was considered statistically significant as long as the results were consistent with that of sensitivity analyses. For secondary analyses a *p*-value lower than a Benjamini and Hochberg corrected significance level was considered significant. The analysis was performed using Statistica Software version 13.3 (StatSoft; Tulsa, OK, USA) and R Software version 4.0.0 with package “mice” version 3.8.0 (R Foundation for Statistical Computing; Vienna, Austria). The underlying raw data has been made publicly available through the Mendeley Data repository (http://dx.doi.org/10.17632/dzbr42ddrm.1).

## Results

### Study Participants

Sixty healthy volunteers were enrolled to the original study (35) and randomly allocated to the groups of *Sb* and placebo. Of these, seven (12%) dropped out before the intervention started and another two (3%) were lost during the intervention period. Out of 51 participants who completed the study, salivary 5-HT concentration was determined in a group 32 (63%) people. The group comprised equal numbers of men and women and their mean age was 22.8 ± 1.7 years: 16 people were in the *Sb* group and 16 in placebo. Sociodemographic and basal psychological, biochemical and physiological characteristics were balanced between the study groups apart from the sex: more men were in the *Sb* group than in the placebo group (**Table 1**).

**Table 1:** Sociodemographic and basal psychological, biochemical and physiological characteristics of the study participants for whom salivary serotonin concentration was determined.

Characteristics	Total sample (n=32)	Study group		Comparison of the study groups^a^
		Placebo (n=16)	*Saccharomyces* (n=16)	
**Sociodemographic**				
Sex (male) ^b^	16 (50%)	5 (31%)	11 (69%)	2.20
Age years ^c^	22.8 (1.7)	23.0 (1.6)	22.6 (1.7)	0.98
**Psychological**				
Eating attitudes ^d,e^	7 (3-14)	7.5 (3-14)	7 (3-15)	0.93
Depression ^d,f^	6.5 (2.5-9)	8 (3.5-9)	5.5 (2-9)	0.69
Perceived stress ^d,g^	15.5 (9-22.5)	16.5 (8-23)	13.5 (9-20.5)	0.82
Trait anxiety ^d,h^	38 (32.5-48.5)	38 (33-46)	39 (29.5-48.5)	1.03
State anxiety ^d,i^	33.5 (29-40.5)	33.5 (28.5-45)	33.5 (29.5-40)	1.00
**Biochemical**				
Salivary cortisol ng × mL^-1 d^	1.96 (1.36-3.63)	2.14 (1.58-3.68)	1.61 (1.33-3.04)	0.75
Salivary metanephrine pg × mL^-1 c^	33.3 (18.1)	33.2 (18.1)	33.5 (18.8)	1.01
**Physiological**				
Body-mass index kg × m^-2 c^	22.8 (2.9)	22.3 (2.8)	23.3 (2.9)	1.04
Pulse rate min^-1 d^	69 (64-82)	75 (64-85.5)	68 (64-75)	0.91

a Saccharomyces-to-Placebo relative proportion for sex and ratio of point estimates for other characteristics, ^b^number and frequency, ^c^mean and standard deviation, ^d^median with 1^st^ and 3^rd^ quartiles, ^e^symptoms and concerns characteristic of eating disorders measured with Eating Attitudes Test-26, ^f^severity of depressive symptoms measured with Beck Depression Inventory, ^g^extent of perceived stress measured with Perceived Stress Scale-10, ^h^trait anxiety measured with the respective part of the State-Trait Anxiety Inventory, ^i^state anxiety measured with the respective part of the State-Trait Anxiety Inventory.

### Salivary Serotonin in the Study Groups

**Figure 3:**
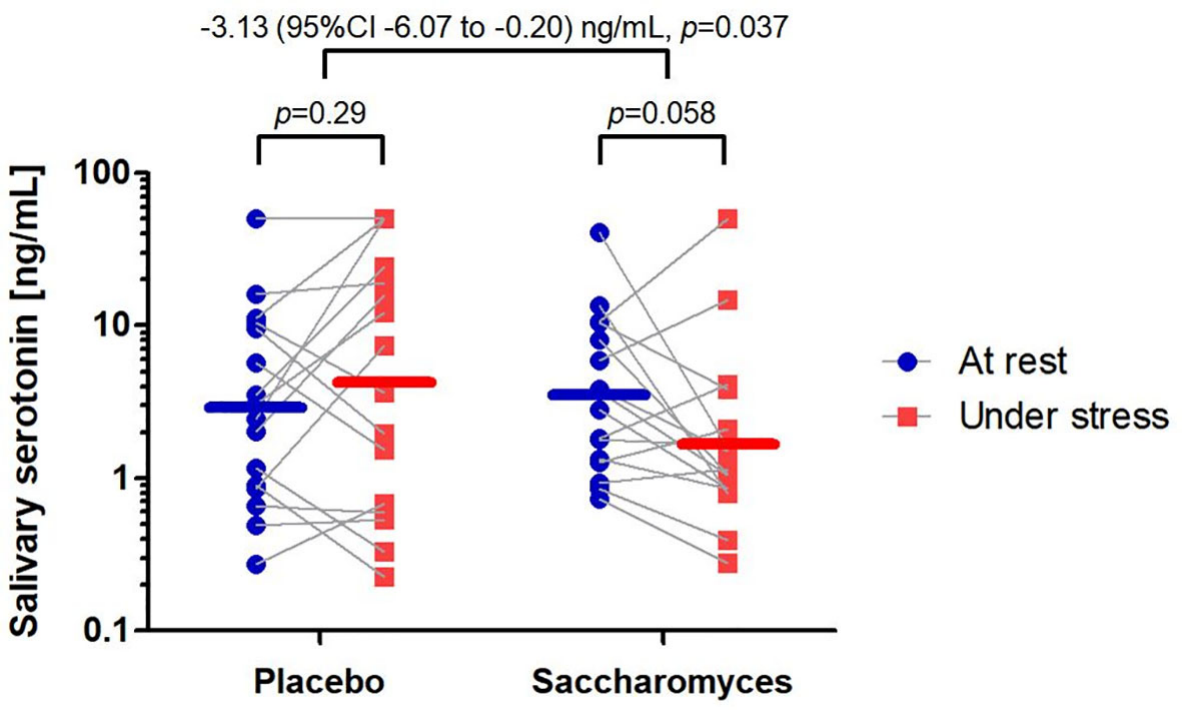
Alteration in salivary serotonin concentration between the study groups. Individual data points are marked and linked with grey lines indicating the shift from “at rest” to “under stress”. The mean concentrations are represented by horizontal lines for both time points. The effect size of the difference in the measures together with *p*-values of *per protocol* analyses are reported above the graph.

According to the PP analysis, the difference in salivary 5-HT level between “under stress” and “at rest” conditions was significantly more negative in *Sb* group than placebo by 3.13 (95% CI 0.20 to 6.07) ng/mL: two-way within-between interaction *F*(1,30) = 4.75, *p* = 0.037 (**Figure 3**). Both ITT analyses were in line with the PP analysis: *F*(1,49) = 8.97, *p* = 0.0043 and *F*(1,58) = 5.34, *p* = 0.024 for mITT and tITT, respectively. The result of PP analysis adjusted for sex, the highly imbalanced baseline parameter (56), was also significant: *F*(1,29) = 4.55, *p* = 0.041.

Analyzing each study group separately, the salivary 5-HT concentration in the *Sb* group tended to decrease from “at rest” (3.45, 95% CI 1.85 to 6.41 ng/mL) to “under stress” (1.75, 95% CI 0.88 to 3.50 ng/mL) by 1.69 (95% CI -0.07 to 3.46) ng/mL (one-way repeated-measures ANOVA *F*(1,15) = 4.19, *p* = 0.058). In the placebo group, no significant change in salivary 5-HT concentration was observed from “at rest” (2.87, 95% CI 1.33 to 6.31 ng/mL) to “under stress” (4.31, 95% CI 1.55 to 11.97 ng/mL); the difference between the time points in placebo group was 1.44 (95% CI -1.36 to 4.24) ng/mL (one-way repeated-measures ANOVA *F*(1,15) = 1.20, *p *= 0.29). The findings in the placebo group may suggest negligible effect of psychological stress alone on salivary 5-HT or the effect reversed by placebo response.

### Salivary Serotonin and Characteristics of the Study Participants

The PP analysis identified a significant positive correlation of salivary 5-HT concentration with salivary metanephrine level, as well as with pulse rate. This was confirmed by adjusted and both ITT analyses. Assumption of normal distribution of residuals appeared to be met. The PP analysis of the interactions between study time point and salivary 5-HT in predicting salivary metanephrine and pulse rate resulted in no significant effect (*F *(1,59) = 0.53, *p * = 0.47 and *F *(1,59) = 0.02, *p * = 0.88, respectively), what suggests similar effect sizes for the “at rest” and “under stress” associations. Moreover, salivary 5-HT concentration “at rest” closely predicted the level “under stress”, which implies relatively low intra-individual salivary 5-HT variability. No sociodemographic, psychological, or other biochemical and physiological characteristics were significantly correlated with salivary 5-HT concentration in the PP analyses (**Table 2**).

**Table 2:** Correlations of sociodemographic, psychological, biochemical and physiological characteristics of the study participants with salivary serotonin concentrations “at rest” and “under stress”.

Characteristics	β (95% confidence intervals), *p*-value^a^		
	Salivary 5-HT “at rest” ^b^		Salivary 5-HT “under stress” ^b,c^
**Sociodemographic**			
Sex (0=female, 1=male)	0.07 (-0.31 to 0.44), *p* = 0.72		0.10 (-0.30 to 0.49), *p* = 0.62
Age	-0.15 (-0.52 to 0.22), *p* = 0.40		-0.13 (-0.52 to 0.26), *p* = 0.50
**Psychological**			
Eating attitudes ^d^	0.00 (-0.38 to 0.38), *p* = 0.98		0.03 (-0.33 to -0.40), *p* = 0.86
Depression ^e^	0.09 (-0.28 to 0.46), *p* = 0.63		0.00 (-0.37 to 0.37), *p* > 0.99
Perceived stress ^f^	0.00 (-0.37 to 0.38), *p* = 0.99		0.08 (-0.28 to 0.45), *p* = 0.65
Trait anxiety ^g^	0.01 (-0.36 to 0.38), *p* = 0.97		0.04 (-0.32 to 0.41), *p* = 0.82
State anxiety ^h, i^	0.10 (-0.13 to 0.34), *p* = 0.39 ^c, i^		
Subject knowledge ^j^	-0.13 (-0.50 to 0.24), *p* = 0.47		-0.08 (-0.41 to 0.37), *p* = 0.69
Subject knowledge retrieval under stress ^k^	0.18 (-0.13 to 0.49), *p* = 0.24		0.16 (-0.18 to 0.50), *p* = 0.34
**Biochemical**			
Salivary cortisol ^b, i^	-0.02 (-0.28 to 0.24), *p* = 0.91 ^c, i^		
Salivary metanephrine ^i^	**0.27 (0.02 to 0.52), *p* = 0.031** ^c, i, l^		
Salivary 5-HT “at rest” ^b^	N/A		**0.58 (0.26 to 0.91), *p* = 0.0009**
**Physiological/anthropometry**			
Body-mass index	0.05 (-0.33 to 0.42), *p* = 0.80		0.00 (-0.37 to 0.37), *p* = 0.98
Pulse rate ^b, i^	**0.28 (0.05 to 0.50), *p* = 0.018** ^c, i, m^		

The results presented in bold are statistically significant at the Benjamini and Hochberg corrected significance level of 0.039 (false discovery rate = 0.3).

5-HT—5-hydroxytryptamine (serotonin).

N/A, not applicable.

a per protocol linear regression analysis, ^b^log-transformed to bring the distribution closer to normal, ^c^analyses adjusted for Saccharomyces intake, ^d^symptoms and concerns characteristic of eating disorders measured with Eating Attitudes Test-26, ^e^severity of depressive symptoms measured with Beck Depression Inventory, ^f^extent of perceived stress measured with Perceived Stress Scale-10, ^g^trait anxiety measured with the respective part of the State-Trait Anxiety Inventory, ^h^state anxiety measured with the respective part of the State-Trait Anxiety Inventory, ^i^data pairs for “at rest” and “under stress” conditions were combined in each analysis (n = 64) to increase statistical power, ^j^measured as the score in Pharmacology pre-examination test adjusted for the faculty to control for the variability between the test sets, ^k^measured as the number of points in the final examination in Pharmacology adjusted for Pharmacology pre-examination test score and examination set to control for the subject knowledge and variability between the examination sets, ^l^following adjustment for sex, age, body-mass index and depression: p = 0.023, modified intention to treat analysis: p = 0.0032, true intention to treat analysis: p = 0.0003, ^m^following adjustment for sex, age, body-mass index and depression: p = 0.013, modified intention to treat analysis: p = 0.021, true intention to treat analysis: p = 0.0053.

## Discussion

The anti-depressant, anti-anxiety and pro-cognitive potential of bacterial probiotic supplementation has been tested in numerous pre-clinical (17) and clinical studies (18–20, 22). It has also been proposed that these properties may be associated with 5-HT (20, 23). In contrast, however, scarce research exists on yeast psychobiotics in this area. The present study appears the first to demonstrate that administration of the yeast probiotic *Sb* decreases salivary 5-HT under psychological stress in humans as compared to placebo. Furthermore, it shows salivary 5-HT to be positively associated with sympathoadrenal markers, but not with the psychologically-relevant characteristics.

For almost a century, 5-HT and *Sb* have been known to demonstrate opposed effects on gastrointestinal peristalsis, with 5-HT promoting it and *Sb* inhibiting it. 5-HT was initially extracted from EC cells of the gastrointestinal tract and found to cause smooth muscle contraction (2). *Sb* was accidentally discovered during a cholera outbreak as an ingredient of fermented anti-diarrheal tea (33). However, 5-HT and *Sb* were only recently directly linked to each other. In a randomized controlled trial by Gu et al. administration of *Sb* culture supernatant to mice was found to reduce intestinal motility and up-regulate serotonin transporter (SERT) at the mRNA and protein level (58). This likely led to 5-HT cellular influx and enhanced degradation (5) as the *Sb* supernatant-treated mice exhibited lower concentrations of 5-HT in intestinal tissue (58). Such decrease in 5-HT level in the gut could prevent transport of the neurohormone to the blood and block its subsequent move to saliva (59), as observed in the present study.

The finding that *Sb* administration decreases salivary 5-HT contradicts most studies on bacterial probiotics (17–20). In fact, *Sb* is a distinct probiotic, being not bacterial but fungal (32, 33). These two groups substantially differ in terms of biological characteristics, mechanism of action and beneficial properties (36, 37). Consequently, it is unlikely that the effects of bacterial probiotics can be directly extrapolated to fungal ones (35).

Metanephrine is a chemically stable metabolite of epinephrine (60), which is known as a key enhancer of heart rhythm (61). Metanephrine is released to the blood circulation and easily transported to saliva (35, 62, 63). Its salivary level together with pulse rate may be considered biomarkers of symapathoadrenal activity (60, 64). In the present study, we tested correlations of salivary 5-HT concentration with several parameters. We found both discussed symapathoadrenal markers positively correlated with salivary 5-HT concentration. These findings are supported by literature data linking 5-HT with epinephrine. Rats presented a dose-dependent increase in plasma epinephrine levels in response to peripherally administered 5-HT (65). Similar effects resulted from infusion of a 5-HT_1A_ receptor agonist into the paraventricular nucleus of the hypothalamus in rats, however, norepinephrine concentration was not affected (66). In a human study of hyper- and normotensive volunteers, plasma and platelet 5-HT concentrations were found to exhibit low to moderate positive correlation with pulse rate, but not with systolic or diastolic blood pressure (67).

As we found *Sb* to reduce salivary 5-HT, and salivary 5-HT level to positively link with heart rate, it cannot be easily concluded that *Sb* reduces heart rate. In fact, our original study has reported the opposite effect (35). Such apparent contradiction is possible: positive correlations of A with B and B with C allows for a negative correlation between A and C (68) (p. 255). It may be suggested that an elevated heart rate caused by *Sb* under stress, which was observed in our original study (35), is independent of the effect of *Sb* on salivary 5-HT level as reported in the current study.

In the present study, we examined several psychologically-relevant characteristics, including anxiety symptoms and markers, depressive symptoms and eating attitudes, as well as cognitive-related academic examination performance. Intriguingly, none of the characteristics were found to significantly correlate with salivary 5-HT concentration. Yet 5-HT is perhaps the best known modulator of multiple neuropsychological processes (1). This incompatibility may be partially explained by the fact that most applied psychometric tests covered a wide time frame, which may not be captured by a single time-point assessment of salivary 5-HT. Another explanation can be attributed to the site of origin of 5-HT in human saliva, which appears to be peripheral rather than CNS, as outlined in the majority of relevant studies (24, 25, 30, 31, 69). The only studies suggesting the level of 5-HT in saliva reflects its CNS status offer modest support: they either report a relatively low effect size (26) or examine diurnal fluctuations in salivary 5-HT (27). On the other hand, lack of statistically significant finding is not a proof of “no association at all” and may arise from inadequate sample size, particularly as sample size for the present study was estimated based on potential serotonergic effect of *Sb* supplementation. Thus, no firm conclusion can be drawn from the current results regarding salivary 5-HT link to psychological functioning.

Finally, the matter of salivary 5-HT concentration itself requires some attention. In the present study, the mean salivary 5-HT concentrations were found to be similar to those reported elsewhere in healthy young adults (26, 27). Slightly higher salivary 5-HT concentrations have been reported in children evaluated for neurological diseases (24) and dogs (31). On the other hand, much lower salivary 5-HT concentrations have been reported in young adult phenylketonuria patients (25), and much higher ones in other young adults (30, 70) with the differences being up to a hundredfold. The high inter-study variability in salivary 5-HT level may result from differences in the preparation of study participants for salivary collection. It is known that nuts, pineapple and bananas, and some other foods, contain 5-HT and Trp (50, 51), and participants in the present study were instructed to avoid their consumption a dozen hours before sampling; however, other studies were not as stringent (26, 30) or the rules were not reported (25, 70). Moreover, some dietary effects on 5-HT level appear unavoidable in research settings as carbohydrate-rich meals increase plasma 5-HT while protein-rich meals lower plasma 5-HT (71), with salivary 5-HT likely changing accordingly (59). Similarly, physical activity affects salivary 5-HT level (30); the participants in the present study were therefore requested to avoid exercise on the day of salivary collection, as in some other studies (27), but not all. In addition, participant characteristics (72), collection time (27) and collection method (59) significantly influence 5-HT levels and may account for the abovementioned variability. This observed variation in salivary 5-HT concentrations between studies challenges the comparability of research results and demands further explanation.

The present study has several limitations. First of all, it is a secondary analysis of a randomized controlled trial and as such, the results should be interpreted with caution (38, 39) as potentially presenting type I error. This is particularly the case as significant *p*-values for *per protocol* analyses were not much lower than statistical significance threshold. Secondly, 5-HT was only studied in saliva. Although this source may be relevant to research on gut microbiota and probiotics (31), it would be interesting to test other body fluids for 5-HT and its metabolites in order to capture broader picture of the serotonergic effect of *Sb*. It cannot be also concluded whether the current result of *Sb*-induced decrease in salivary 5-HT is relevant to central serotonergic neurotransmission. Importantly, central and peripheral 5-HT plays different biological roles, and future research should take this into account (28, 29). Thirdly, the effect of *Sb* supplementation was examined only in the context of psychological stress; it was decided to not additionally investigate the *Sb* effect in the settings without psychological stress due to logistical reasons and the risk of inflated participants' dropout rate. As a result, it remains unknown whether the reported effect on salivary 5-HT results from the *Sb* intake alone or its interaction with stress. This shortcoming of the study design may be addressed in a follow-up research. Fourthly, some of the measures correlated to salivary 5-HT may have limited validity. Pulse rate was self-recorded manually, which may generate biased results, particularly under stress (73, 74). Cognitive functioning was modeled with academic examination performance, assessed as retrieval of subject knowledge under stress (examination score in relation to test score performed in non-stressful condition) and subject knowledge itself. The former cognitive characteristic, however, appears to involve more than just information recall: the test anxiety phenomenon, which tends to reduce examination achievements, is additionally characterized by easy distraction, loss of coherent thoughts and difficulties in reading and understanding questions (75). Hence, the reported names of constructs may not be fully accurate. Fifthly, 5-HT concentration was not measured in all the available salivary specimens. This, however, should not be regarded as bias-generating due to random pattern of sample loss. The only problem it could have resulted is diminished statistical power with a potential for type II error due to decreased sample size. Lastly, the present findings should not be generalized beyond the examined probiotic strain of *Sb* CNCM I-1079 and even its current technological form (76, 77).

## Conclusions

According to our findings, healthy volunteers supplementing their diet with *Saccharomyces boulardii* CNCM I-1079 for 30 days present decreased concentration of salivary 5-HT under psychological stress as compared to placebo; this remains unknown whether such supplementation affects the outcome with no influence of psychological stress. Moreover, salivary 5-HT in healthy young adults appears positively correlated with symapathoadrenal markers. Although the obtained results are supported by the literature, they should be regarded with caution. Further studies are needed to confirm the findings, to determine their possible clinical relevance and to explain whether salivary 5-HT may serve as a valid biomarker in scientific and clinical investigation.

## Data Availability Statement

The dataset analyzed for this study can be found in the Mendeley Data repository under the following link: http://dx.doi.org/10.17632/dzbr42ddrm.1.

## Ethics Statement

The study was approved by the Bioethics Committee of the Medical University of Lodz (RNN/86/16/KE, received on 19 April 2016). Written informed consent was obtained from all participants upon entrance to the study.

## Author Contributions

Conceptualization MK. Methodology MK. Software N/A. Validation MK and EK. Formal analysis MK. Investigation MK, AW-O, NS, PK, and MC. Resources MK and JK. Data curation MK. Writing—original draft preparation MK, JK, and AW-O. Writing—review and editing MK, JK, AW-O, PK, MC, NS, TP, and EK. Visualization MK. Supervision EK and TP. Project administration MK. Funding acquisition MK and EK. All authors have read and agreed to the published version of the manuscript.

## Funding

The paper has been supported by the Medical University of Lodz with the grant for young scientists no. 502-03/5-108-03/502-54-157 (received by MK) and the grant no. 503/5-108-03/503-51-001-19-00 (received by EK).

## Conflict of Interest

The authors declare that the research was conducted in the absence of any commercial or financial relationships that could be construed as a potential conflict of interest.

## Publisher’s Note

All claims expressed in this article are solely those of the authors and do not necessarily represent those of their affiliated organizations, or those of the publisher, the editors and the reviewers. Any product that may be evaluated in this article, or claim that may be made by its manufacturer, is not guaranteed or endorsed by the publisher.
